# Prenatal hyperbaric normoxia treatment improves healthspan and regulates chitin metabolic genes in *Drosophila melanogaster*

**DOI:** 10.18632/aging.101084

**Published:** 2016-10-24

**Authors:** Suyeun Yu, Eunil Lee, Bodokhsuren Tsogbadrakh, Gwang-Ic Son, Mari Kim

**Affiliations:** ^1^ Department of Preventive Medicine, College of Medicine, Korea University, Seoul, 136-701, Republic of Korea; ^2^ Department of Internal Medicine, Seoul National University Hospital, Seoul, 151-742, Republic of Korea

**Keywords:** aging, healthspan, hormesis, hyperbaric normoxia, development, chitin metabolism, *Drosophila melanogaster*

## Abstract

Aging is a universal, irreversible process accompanied by physiological declines that culminate in death. Rapid progress in gerontology research has revealed that aging can be slowed through mild stress-induced hormesis. We previously reported that hyperbaric normoxia (HN, 2 atm absolute pressure with 10% O_2_) induces a cytoprotective response *in vitro* by regulating fibronectin. In the present study, we investigated the hormetic effects of prenatal HN exposure on *Drosophila* healthspan related to molecular defense mechanisms. HN exposure had no disruptive effect on developmental rate or adult body weight. However, lifespan was clearly enhanced, as was resistance to oxidative and heat stress. In addition, levels of reactive oxygen species were significantly decreased and motor performance was increased. HN stress has been shown to trigger molecular changes in the heat shock response and ROS scavenging system, including *hsp70*, *catalase*, *glutathione synthase*, and *MnSOD*. Furthermore, to determine the hormetic mechanism underlying these phenotypic and molecular changes, we performed a genome-wide profiling in HN-exposed and control flies. Genes encoding chitin metabolism were highly up-regulated, which could possibly serve to scavenge free radicals. These results identify prenatal HN exposure as a potential hormetic factor that may improve longevity and healthspan by enhancing defense mechanisms in *Drosophila*.

## INTRODUCTION

All living systems have the intrinsic ability to respond to external and internal stressors and to maintain homeostasis [1, 2]. A molecular homeodynamic balance increases the efficiency of multiple pathways, including nuclear and mitochondrial DNA repair, detoxification and immune and stress responses [3]. A failure in the homeodynamic pathway culminates in death because of aging due to accumulated molecular and cellular damage [2, 4, 5].

The phenomenon that low doses of potentially toxic substances can induce a beneficial biological response is termed hormesis [6-9]. These beneficial low doses, called mild stress, include a wide variety of biological, chemical, and mechanical stimuli. Accumulating studies have established a strong link between hormesis and aging, as a result of overcompensation of defense mechanisms induced by mild stress. For example, exposure to mild stresses such as irradiation, heat, or hypergravity improves mean lifespan in *Drosophila melanogaster* [10-13] and improved stress tolerance in *D. melanogaster* [12, 14] and in *Caenorhabditis elegans* [15].

We previously reported that hyperbaric normoxia (HN, 2 atmospheres absolute with 10% O_2_) as a mechanical mild stressor shows hormetic effects, such as inducing a cytoprotective response *in vitro* by regulating fibro-nectin expression and DNA damage [16]. Mechanical stress caused by stretching, compression, fluid shear stress, or hydrostatic pressure is the primary regulator to induce homeostatic mechanisms. It has been shown to influence major developmental processes, such as in skeletal muscle, bone, cartilage, blood vessels and heart, and to affect diverse cellular processes including cellular growth, differentiation, migration, apoptosis, and senescence [17, 18]. Mechanical stress has been well studied in development research [19], but its application to biogerontologic research as a hormetic factor remains unclear.

There is growing evidence to suggest that exposure to environmental stress during critical periods of development result in permanent effects on behavior and metabolism, as well as growth, reproduction, stress tolerance and lifespan [20-22]. These observations were explained by the concept of developmental plasticity [23], which causes genetic or epigenetic modulation. In particular, the hormetic effects of dietary regulation on developmental plasticity have been investigated in life-long health studies across many different animal species [24-27]. The effects of hormesis and its application to biogerontologic research are being explored, and one of the next challenges is to identify new mild stresses that have hormetic effects at the organismal level.

Hyperbaric normoxia stress shows no genotoxicity on various cell lines, and in fact shows cytoprotective effects [16]. However, evidence that these hormetic effects of HN also occur *in vivo* is lacking. In the present study, using *D. melanogaster* as a model system we investigated the effect of prenatal HN exposure on healthspan, including developmental rate, fecundity, severe stress tolerance, longevity and behavioral aging. In addition, we addressed the molecular consequences of HN, as a mec-hanical mild stress in response to the relevant aging trait.

## RESULTS

### Characterization of HN in *D. melanogaster* development

**Figure 1:**
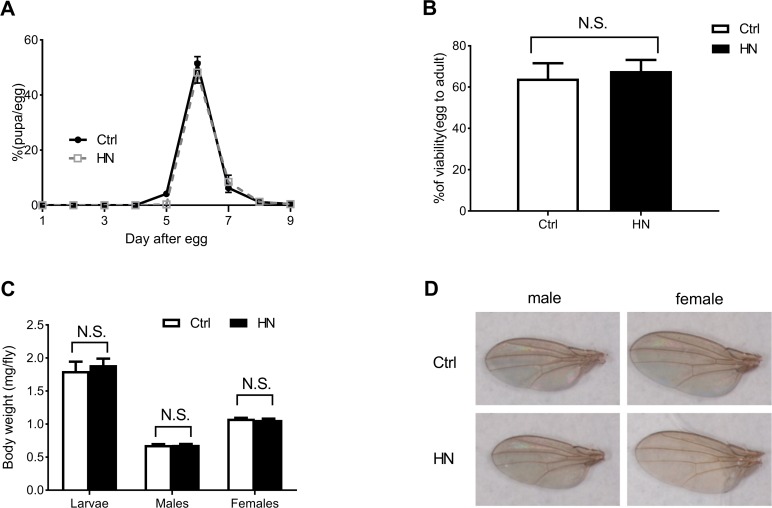
The effects of HN on growth pattern and rate (**A**) HN-treated larvae pupariated at normal time points and were slightly delayed at 5day after egg laying (>80 animals/group). (**B**) The egg-to-adult viability was no significant difference between control and HN exposure (>60 eggs/group, n≥5 independent experiments). (**C**) Average weight of larvae (Ctrl, n=187; HN, n=192) and adult flies (Ctrl male, n=53; HN male, n=54; Ctrl female, n=41; HN female, n=46) indicated. (**D**) HN-treated flies had no significant change in wing size (5 flies/group, n≥3). Error bars denote the standard error of the mean (SEM). NS: not significant.

To determine the effects of HN in *D. melanogaster*, we examined whether prenatal HN exposure affected development at each stage of growth. We observed no significant effects of HN on growth compared to controls. Although the timing from larva to pupa was delayed at day 5 (*p* =0.003), the total larval growth pattern, total development time from egg to eclosion (Fig. 1A) and total average eclosion rate (egg to adult survival) did not differ between HN and control groups (Fig. 1B). Moreover, HN did not affect the mean body weight of males, females or larvae at eclosion (Fig. 1C) or wing size (Fig. 1D).

### Protection against heat and oxidative stresses by prenatal HN exposure

**Figure 2:**
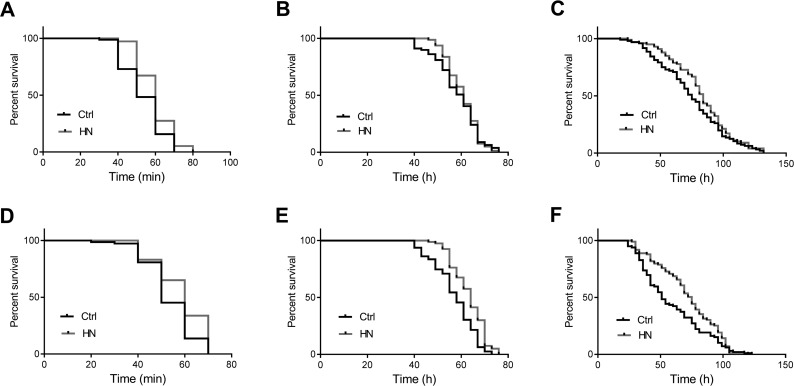
Enhanced heat and oxidative stress tolerance by prenatal HN exposure in both sex HN exposure led to significantly increased resistance to heat stress in male (**A**) *p* =0.0001; female flies (**D**) *p* =0.021 and oxidative stress (**C**) male, *p* =0.031; (**F)** female, *p* =0.0004. HN exposure affected starvation resistance in female (**B**) *p* <0.0001) but not in male flies (**E**) *p* =0.2004). HN-treated flies presented a significant increased (black: controls; grey: HN exposure).

We previously showed that hyperbaric normoxia protects normal fibroblasts (WI-38) against oxidative stress [16]. We therefore examined whether HN treatment also enhanced stress tolerance in *D. melanogaster* (Table 1A). To determine the tolerance to oxidative stress, flies were fed 18 mM paraquat added to standard food and their survival was monitored. We found that for both male and females, HN-treated flies exhibited a significant resistance to oxidative stress, 12% increase of median survival in males (Fig. 2C) and 47% in females (Fig. 2F), whereas control flies exhibited high sensitivity to paraquat. Under 40°C heat stress, both male and female HN-treated flies showed increased thermal tolerance by 20% in both males (Fig. 2A) and females (Fig. 2D). In addition, when subjected to nutrient deprivation, only female flies showed increased resistance to starvation, compared to their controls (males, Fig. 2B; females, Fig. 2E). These results suggest that HN plays a protective role against different stresses in *Drosophil*a, which is in agreement with previous *in vitro* reports [16].

**Table 1A:** Summary of survival analysis Altered stress tolerance and lifespan by prenatal HN exposure altered stress tolerance.

*w^1118^*	sex	Median survival time (hour)				ILS	*p-*value
		Ctrl	(n)	HN	(n)		
Heat	M	0.83	(90)	1	(114)	1.20	0.0001
	F	0.83	(74)	1	(84)	1.20	0.007
Starvation	M	61	(80)	61	(80)	1.00	0.2004
	F	58	(80)	64	(80)	1.10	<0.0001
Paraquat	M	75	(96)	84	(98)	1.12	0.0313
	F	51	(100)	75	(100)	1.47	0.0004

Mean lifespan (MLS), maximum lifespan (MaxLS), and increased lifespan (ILS, ratio of HN to Control) are presented with the number (n) of flies tested.

**Table 1B:** Summary of survival analysis Altered stress tolerance and lifespan by prenatal HN exposure altered lifespan.

Sex	Ctrl			HN			ILS	*p-*value
	MLS	MaxLS	(n)	MLS	MaxLS	(n)		
males	45.5	78	(236)	51	83	(226)	1.12	<0.0001
females	49	72	(224)	56	75	(224)	1.14	<0.0001

Mean lifespan (MLS), maximum lifespan (MaxLS), and increased lifespan (ILS, ratio of HN to Control) are presented with the number (n) of flies tested.

### Enhanced longevity and healthspan by hyperbaric normoxia

**Figure 3:**
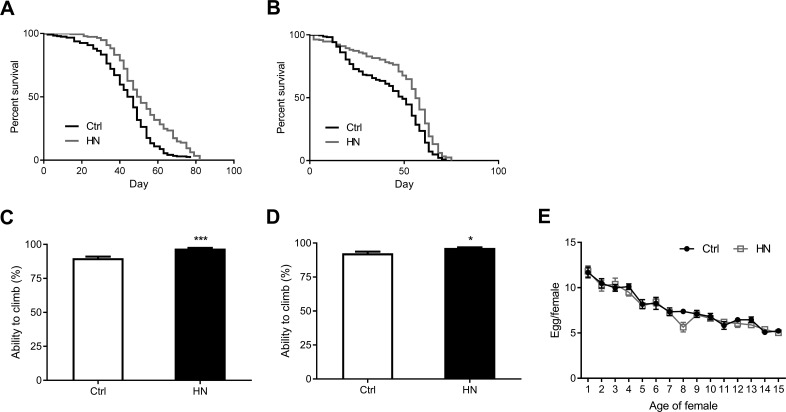
Increased healthspan in HN-treated flies HN exposure led to increased mean lifespan by 12% in males (**A**) *p* <0.0001) and by 14% in females (**B**) *p* <0.0001. HN-treated flies showed a significant higher climbing performance then control groups (**C**) male, *p* =0.0007; (**D**) female, *p* = 0.028. (**E**) HN exposure did not affect the fecundity while there was decreased fertility at day 8 (*p* =0.009). Error bars denote SEM. Values with different superscripts are significantly different at *p* <0.05 (* = *p* <0.05, ** = *p* <0.01, *** = *p* <0.01).

To gain further insight into the protective effects of HN, we assayed the lifespan in treated and untreated flies (Table 1B). We found that the median lifespan of HN-treated flies was increased by 12% in males (Fig. 3A) and by 14% in females (Fig. 3B). Motor performance is regarded as a marker of healthspan in *D. melanogaster*. [28, 29]. To measure this, we tested the vertical climbing ability of treated and untreated flies. We found that HN treatment improved the flies' ability to reach the top of the vial (males, Fig. 3C; females, Fig. 3D), indicating an improved motor performance. We also tested reproductive output, which is often negatively correlated with extended lifespan and healthspan [30, 31]. However, our results showed that while HN-treated female flies showed decreased fertility at day 8, there was no detrimental effect on average daily fecundity compared to controls (Fig. 3E). This implies that prenatal HN exposure has the ability to delay the physiological age without any associated reproductive costs.

### Changes in ROS production and oxidative stress-related gene expression

**Figure 4:**
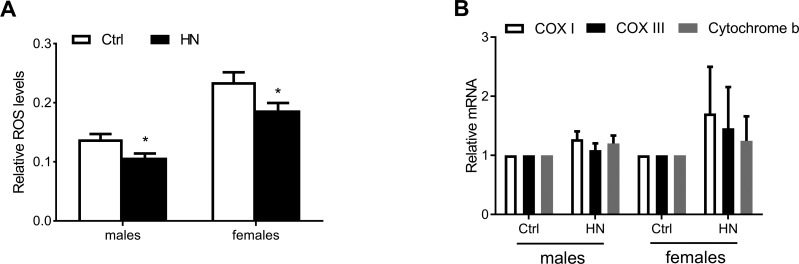
Decreased ROS production by HN exposure (**A**) ROS generation was reduced in HN-treated flies (male, *p* =0.013; female, *p* =0.035). (**B**) The expression levels of mitochondrial DNA were not changed in both sex. Error bars denote SEM. * = *p* <0.05, ** = *p* <0.01, *** = *p* <0.01.

**Figure 5:**
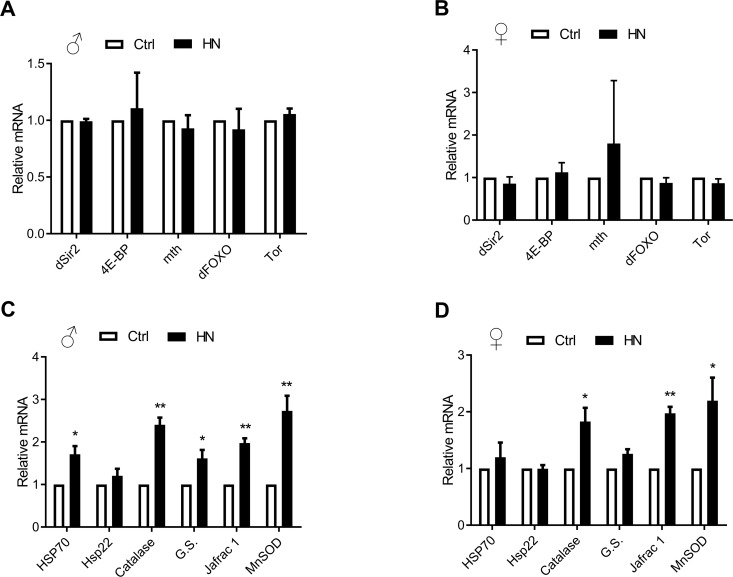
Altered oxidative stress-related gene expression by HN exposure (**A**-**B**) Aging related gene levels were not changed. (**C**-**D**) HN-treated mRNA expression related with oxidative stress genes were increased in both sex. Error bars denote SEM. * = *p* <0.05, ** = *p* <0.01, *** = *p* <0.01.

Aging leads to increasing ROS as well as impaired motor performance associated decreased lifespan [32]. The relationship between ROS and healthspan has been studied extensively. In the present study, we examined ROS levels in HN-treated flies, using a FOX assay. Compared to control flies, the average amount of ROS in HN-treated flies was decreased by 10% for both males and females (Fig. 4A). Otherwise, there was no difference in expression levels of mitochondrial-encoded genes, such as *cytochrome c oxidase subunits I* and *III* (*COXI*, *COXIII*) and *cytochrome b* (*Cytb*) in males or females (Fig. 4B). To test if ROS reduction by HN exposure leads to altered gene expression associated with aging and oxidative stress, we monitored mRNA levels of candidates. We analyzed the gene expression level of *Drosophila methushelah (mth)* and *Sir2*, *4E-BP*, *FOXO* and *Tor* in nutrient signaling, there was no changes (males, Fig. 5A; females, Fig. 5B). However, Oxidative stress-related genes including *hsp70*, *catalase*, *glutathione synthase* (*GS*), *Jafrac 1*, and *Superoxide dismutase 2* (*MnSOD*) were up-regulated following HN exposure (males, Fig. 5C; females, Fig. 5D). These data suggest that the prolonged healthspan that results from prenatal HN exposure is associated with anti-oxidant defense mechanisms.

### Global gene expression profiles in HN treated healthspan increased flies

**Figure 6:**
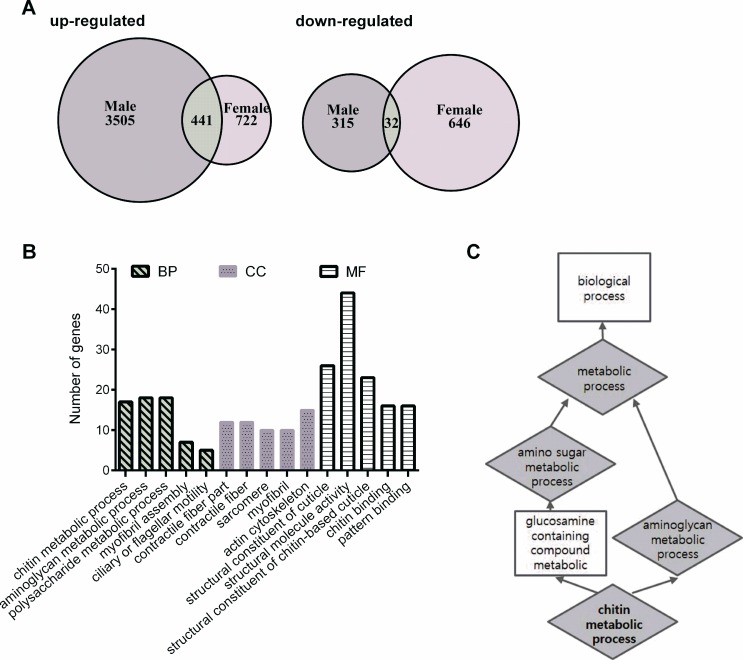
Differential gene-expression analysis and enriched gene ontology categories (**A**) Venn diagram comparing the up-regulated and down-regulated genes in HN-treated flies. (**B**) Annotated genes from the overlap of up-regulated gene lists can be divided into three major categories, including biological process (BP), cellular component (CC), and molecular function (MF). The vertical scale on the left indicated the number of genes in the sub-categories. (**C**) The functional enrichment map were illustrated for a number of biological function including the enriched target genes.

To obtain a global view of the genomic response to HN treatment in the healthy aging phenotype in male and female flies, we performed a microarray using 32,162 probe sets and analyzed it together with its control. Genes with a more than 1.5-fold change (with statistical significance) following HN treatment were presented as Venn diagrams (Fig. 6A). In total, 4,293 genes (3,946 induced; 347 repressed) were altered in males and 1,841 genes (1,163 induced; 678 repressed) were altered in females following prenatal HN exposure. Comparison of these two sets revealed a significant overlap of 473 genes, among which 441 genes were up-regulated in the HN-treated group. These up-regulated genes can be analyzed in Gene Ontology (GO) which is grouped into three major categories, including biological process (BP), cellular component (CC), and molecular function (MF) (Fig. 6B). The top five sub-categories in BP were indicated in aminoglycan metabolic process (18 genes), chitin metabolic process (17 genes), Polysaccharide metabolic process (7 genes), myofibril assembly (7 genes) and ciliary or flagellar motility (5 genes).

### Chitin metabolism is involved in the improved healthspan seen in HN-treated flies

**Figure 7:**
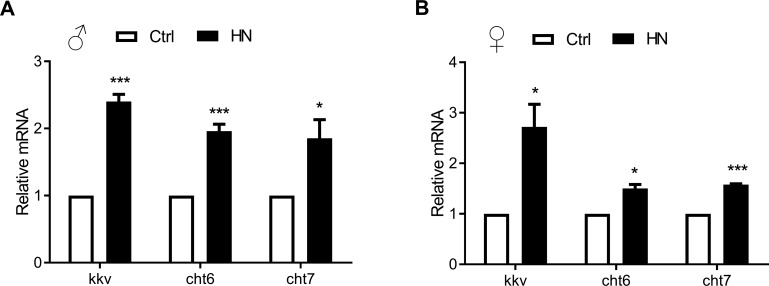
Quantitative real-time PCR validation of HN-treated healthspan genes acquired from microarray analysis Long-lived HN exposure flies increased the expression of 3 genes from the most up-regulated genes among GO terms. Error bars denote SEM. Values with different superscripts are significantly different at *p* <0.05 (* = *p* <0.05, ** = *p* <0.01, *** = *p* <0.01).

Our microarray analysis revealed changes in 18 transcripts involved in aminoglycan metabolic process. Among these, 17 transcripts were found to be involved in chitin metabolism, which is related to aminoglycan meta-bolism according to functional enrichment map. Using KEGG pathway analysis to confirm the over-lapping up-regulation following prenatal HN exposure, amino sugars and nucleotide sugar metabolism were enriched in the HN-treated group. Potential healthspan-promoting genes *Chitinase 6* (*Cht6*: CG43373), *Chitinase 7* (*Cht7*: CG1869) and *kkv*, were selected for further analysis. Validation by qPCR revealed that mRNA levels of *Cht6*, *Cht7*, and *krotzkopf verkehrt* (*kkv*) were highly increased in prenatal HN-exposed flies (males, Fig. 7A; females, Fig. 7B). These observations identify chitin metabolism as a potential molecular mechanism involved the horme-tic effects of HN that result in a prolonged healthspan.

## DISCUSSION

The aims of this study were to test the potential of HN as a mild stressor that can prolong lifespan and healthspan in *Drosophila* and to elucidate the hormetic mechanisms by which HN promotes longevity. While the hormetic effect of exposure to various mild stresses on healthspan and longevity is well documented at adult stages, the potential hormetic effects of prenatal exposure to mild stress remain relatively unknown. Most of the prenatal stress studies were restricted to harmful environmental exposures such as carbon monoxide, alcohol and drugs, which can lead to subvert normal development [33, 34]. In general, prenatal exposure to mild stress has a more pronounced impact on development and the whole life of an organism than postnatal exposure. Therefore, in the present study, before using prenatal HN exposure as a mild stressor, it was necessary to determine any side-effects on the development of *D. melanogaster*. Our data showed that prenatal HN exposure had no detrimental effects on development, such as larval growth rate and embryonic mortality, and body weight of larvae and adults. In addition, prenatal HN exposure did not affect wing size, which is used as an indicator of body size, after growth has stopped. Therefore, our observations indicate that prenatal HN treatment has no deleterious effects on *Drosophila* development.

Mild stress intervention has been shown to improve stress resistance and lifespan, and therefore slow down the aging process [35, 36]. Long-lived mutants often show increased stress resistance to environmental stimuli [35]. Therefore, we hypothesized that HN-induced mild stress would enhance lifespan and stress resistance against critical heat, starvation and oxidative stress. Our results showed that prenatal HN exposure induced longevity and stress resistance to thermal and oxidative stress in both male and female flies. We found no change in starvation resistance, but this could be correlated with a number of other life-history traits, including body size. Thus, our study provides additional independent evidence for the use of HN-induced mild stress as a hormetic effector in *Drosophila*, as was suggested by a previous *in vitro* study [16].

Simply extending lifespan has no value if it is not coupled with improved healthspan. Therefore, sought to determine whether the beneficial effects of prenatal HN exposure could be explained in terms of improved and prolonged lifespan. In *Drosophila*, muscle strength is essential for motor performance and is known to decline with age, causing age-related health problems [28, 29]. Locomotor activity is therefore a reliable marker of healthspan. Indeed, we found that HN exposure improved locomotor activity, indicating an improve-ment in the healthspan of the treated flies. We also tested whether prenatal HN exposure affected female egg-laying ability. Previous diet manipulation studies have reported that an increased lifespan is accompanied by compromised reproduction [30, 31]. The existence of such a trade-off between longevity and fecundity seems reasonable in terms of allocation of resources under food-limited conditions. However, reduced reproduction does not necessarily improve lifespan, and several studies have reported no correlation between longevity and fecundity [37-39]. In the present study, we showed that prenatal HN exposure increased stress tolerance and healthspan without a concomitant reduction in fecundity.

It has been proposed that prenatal HN-induced stress triggers a defense mechanism related to improving longevity and healthspan. The free radical theory of aging suggests that ROS-induced accumulation of damage to cellular macromolecules is a major determinant of lifespan [40]. To this end, we assessed the levels of cytosolic reactive oxygen species (ROS) and found a decrease in cytosolic ROS production following HN treatment. It will be interesting to determine how prenatal HN stress induces the defense mechanism against ROS. One type of defense mechanism involved in hormesis is the heat shock response. Hsp70 is a stress-induced chaperone that regulates lifespan in flies [14, 41], and its expression is reduced with increasing age in *D. melanogaster* [42]. Another type of intervention used to increase healthspan and decrease oxidative stress is the ROS scavenging system. Superoxide dismutase and catalase provide the main enzymatic antioxidative defense in insects. During aging, expression levels of MnSOD are down-regulated in *Drosophila* [43, 44]. MnSOD is localized to the inner mitochondrial space and plays a role in mitochondrial DNA protection by converting superoxide to hydrogen peroxide and oxygen. *MnSOD* knockdown has been shown to reduce oxidative stress resistance and lifespan in flies [45], as well as in a muscle tissue-specific knockdown [46, 47]. Catalase converts hydrogen peroxide to water and oxygen and mitochondria-specific *catalase* overexpression was shown to extend the lifespan of mice [48]. In *Drosophila* aging gut, the *catalase* expression level is improved by rapamycin [49] and catalase activity is diminished during aging in *C. elegans* [50].

Although we obtained some molecular evidence to link antioxidant defense and aging, the underlying mechanism of the healthy aging phenomenon following HN stress is still not clear. We therefore carried out a genome wide microarray analysis, and observed enrichment for genes related to chitin metabolism in the prenatal HN-treated flies. Changes in chitin metabolism-related genes would be predicted to result in an accumulation of chitin. Chitin oligosaccharides are a major component of the inset cuticle and are known to act as potential antioxidants by scavenging ROS [51]. Chitin is synthesized by chitin synthase enzyme coding gene *kkv* and changes in its activity have been related to aging in *D. melanogaster* [52, 53]. Interestingly, the interaction partners for chitin synthases, or chitinases, are unknown in insects [54]. We propose that HN stress acts as a mechanical cue to stimulate chitin metabolism as part of an antioxidative defense mechanism.

In summary, we have identified HN stress as a highly effective hormetic factor. We provide independent evidence for the role of prenatal exposure to HN in lifespan extension and increased stress tolerance without detrimental effects on development in both male and female flies. We also found that prenatal HN exposure enhanced motor performance, indicating an improvement in healthspan as well as lifespan. Interestingly, reproductive ability was not affected by HN stress, which is generally considered a resource trade-off for healthspan extension. Furthermore, HN stress decreased ROS levels by inducing the antioxidant defense mechanism. Considerable overlap between phenotypical and molecular mechanisms was explained using a genome-wide analysis of gene expression profiles. Strong up-regulation of genes involved in chitin metabolism was detected, which could possibly function in the process of scavenging free radicals. These results suggest that prenatal HN exposure is a major hormetic factor that improves longevity and healthspan in *Drosophila* by enhancing antioxidant defense mechanisms. Further analyses will be required to understand how the hormetic effects of HN promote healthy aging in other living systems.

## METHODS

### Fly stocks and maintenance

Wild-type *Drosophila melanogaster w^1118^* were obtained from the Bloomington Drosophila Stock Center (Bloomington, IN, USA) and maintained in a climate-controlled incubator with 25°C and 50 ± 5% relative humidity on 12:12 h light/dark cycle. All flies used in this study were raised on fresh medium containing 6% cornmeal, 3% yeast, 3% sugar, 6% glucose, and 0.6% agar.

### Experimental design for hyperbaric normoxia incubation

All hyperbaric normoxia (HN; 2ATA in normoxic conditions) exposures were carried out in a custom-made animal incubator designed for pressure control [55] without change of temperature (25°C), humidity (50 ± 5%) and under a 12:12 h light/dark cycle. Fertilized eggs from 30 3-day-old pairs were collected in batches of 30 into vials, then exposed to HN until eclosion. After HA exposure all newly born (F1) flies were maintained in a climate-controlled incubator as described above.

### Measuring developmental time, eclosion rate and fecundity

We measured larval development time from egg to pupa (at 24-h intervals), eclosion rate from egg to adult and weight of larvae and adults (male or female). For fecundity, newly eclosed F1 generations of 15 virgin female and 7 male flies were collected and placed into a vial. Flies were transferred to fresh vials every day and the number of eggs laid in a 24-h period was counted for 15 days with ten replicates.

### Body weight and wing size analysis

Body weight of individual larva and adult flies was measured. Flies were reared under the same growth conditions and were age-matched (2 days old) before weighing.

To estimate body size, variation in wing size was measured. The left wing was removed and mounted onto double-sided tape on glass slides and sealed with a cover glass. Images of the wings were taken using a Leica MZ 125 microscope and Leica Application Suite LAS version 3.7.0 software (both from Leica Microsystems).

### Climbing and longevity assays

The climbing assay was performed as previously described by Ganetzky et al. [28] with some modifications. Ten-day-old flies (20 males or 20 females per vial) were collected and placed into a vial (height, 10 cm). After a 1 h recovery period from anesthesia, each vial was gently taped to the bottom. At this point, we counted the number of flies that climbed up to a vertical distance of >7.5 cm within 5 s or 7 s. This procedure was repeated five times. After five trials, the number of flies in each vial was counted. At least 100 flies were used for each condition tested.

Lifespan assay was performed as described previously [56]. In short, 80 male or female flies (1-3 day after eclosion) were cultured in an aging chamber [57]. The number of dead flies was counted every 2–3 days, when fresh food vials were replaced. This procedure was repeated three times.

### Assays for stress resistance of adults

To examine ROS tolerance, flies were placed in a vial containing fresh food and 18 mM paraquat (methyl viologen; Sigma-Aldrich, USA), after wet-starvation in a vial containing 1% agar for 6 h. The number of dead flies was recorded every 3 h until all flies had died. Similarly, starvation resistance was measured by feeding with only water in a vial containing 1% agar (Sigma-Aldrich, USA). Dead flies were counted every 3 h. To test heat tolerance, flies were kept in a 40°C incubator and paralyzed flies were counted every 10 min. Each experiment was performed with 10-day-old flies (20 males or 20 females per vial) with at least three replicates.

### Quantitative real-time PCR analysis

F1 generation flies were frozen in liquid nitrogen and stored at −80°C until analysis. Total RNA was extracted from homogenized whole-body lysates using RNAiso reagent (TAKARA, Japan). After treating with DNase I (Invitrogen, USA), cDNA synthesis was performed using a PrimeScript RT Reagent Kit (TAKARA, Japan). Quantitative real-time PCR was performed using Power SYBR Green (Applied Biosystems, USA) and StepOnePlus Real-Time PCR System (Applied Biosystem, USA). To determine mRNA expression levels for each gene, C_T_ values were normalized to those of rp49. Mean fold induction was calculated from the values from 3-6 independent experiments.

### Quantification of mtDNA

Total DNA was extracted from 10 day old flies from the F1 generation using a G-spin Genomic DNA extraction kit (iNtRON Biotechnology, Korea) and subjected to quantitative real-time PCR as described above. Copy number of mtDNA was estimated using Cox I, Cox III, and Cyt b primers [58], relative to rp49.

### ROS measurement

The rate of ROS production in 20 ten day old flies was measured using a ferric-xylenol orange peroxide assay [59]. Whole bodies were minced in 250 μl distilled water containing aminotriazol (2 mg/ml). Experiments were performed in triplicate. After centrifugation 40 μl of supernatant was mixed with 360 μl FOX reagent (250 μM Fe(NH_4_)2SO_4_·6H_2_O, 25 mM H_2_SO_4_, 4 mM butylated hydroxyl toluene in HPLC grade methanol, 100 μM xylenol orange) containing 100 mM sorbitol (all from Sigma-Aldrich, USA). After incubation at room temperature for 40 min, absorbance of the ferric-xylenol orange complex was estimated at 560 nm using a SpectraMax microplate reader (Molecular Devices, USA).

### Microarray

Total RNA from 20 male and 20 female flies from control and HN-treated groups were collected as described above. For each RNA, the synthesis of target cRNA probes and hybridization were performed using a LowInput QuickAmp Labeling Kit (Agilent Technologies, USA) according to the manufacturer's instructions. Briefly, 25 ng total RNA was added to the T7 promoter primer mix and incubated at 65°C for 10 min. cDNA master mix (5X First strand buffer, 0.1 M DTT, 10 mM dNTP mix, RNase-Out, and MMLV-RT) was added to the reaction mix. Samples were incubated at 40°C for 2 h, and RT and dsDNA synthesis was terminated by incubating at 70°C for 10 min. Transcription master mix was prepared according to the manufacturer's protocol (4X Transcription buffer, 0.1 M DTT, NTP mix, 50% PEG, RNase-Out, inorganic pyrophosphatase, T7 RNA polymerase, and Cyanine 3-CTP). Transcription of dsDNA was performed by adding the transcription master mix to the dsDNA reaction samples and incubating at 40°C for 2 h. Amplified and labeled cRNA was purified on an RNase mini column (Qiagen, USA) according to the manufacturer's protocol. Labeled cRNA target was quantified using an ND-1000 spectrophotometer (NanoDrop Technologies, USA). After checking labeling efficiency, 1650 ng of each cyanine 3-labeled cRNA target was used for cRNA fragmentation by adding 10X blocking agent and 25X fragmentation buffer and incubating at 60°C for 30 min. Fragmented cRNA was resuspended with 2X hybridization buffer and directly pipetted onto an assembled Agilent Drosophila (V2) Gene Expression 4X 44K Microarray. Arrays were hybridized for 17 h at 65°C in an Agilent Hybridization oven (Agilent Technologies, USA). Hybridized microarrays were washed according to the manufacturer's protocol (Agilent Technologies, USA).

### Microarray data acquisition and GO analysis

Hybridization images were analyzed using an Agilent DNA Microarray Scanner (Agilent Technologies, USA) and data quantification was performed using Agilent Feature Extraction software 10.7 (Agilent Technologies, USA). Average fluorescence intensity was calculated for each spot and local background was subtracted. All data normalization and selection of fold-changed genes were performed using GeneSpringGX 7.3.1 (Agilent Technologies, USA). Normalization for the Agilent one-color method was performed: Data transformation, set measurements less than 5.0 to 5.0; Per Chip, normalize to 50th percentage. Averages of normalized ratios were calculated by dividing the average of control normalized signal intensity by the average of test normalized signal intensity.

Functional annotation of genes was performed according to DAVID annotation tools [60, 61]. Default DAVID parameters were used. Venn diagrams from two gene list (male and female) in microarray data analysis were created by R software package. All raw data were deposited in the NCBI Gene Expression Omnibus under accession number GSE84069.

### Statistical analyses

Statistical analyses were conducted using PRISM version 6 (GraphPad Software, Inc., USA). All data except survival curves were presented as mean ± SEM and were statistically evaluated using Student *t*-test distribution. Results of lifespan and stress resistance studies were analyzed using the Kaplan-Meier survival test. Comparisons among groups were conducted using Gehan's Wilcoxon test.

## References

[1] Holliday R (2007). Aging: the paradox of life: why we age.

[2] Rattan SI (2006). Theories of biological aging: genes, proteins, and free radicals. Free Radic Res.

[3] Rattan SI (2008). Hormesis in aging. Ageing Res Rev.

[4] Holliday R (1995). Understanding ageing.

[5] Rattan SI, Clark BF (2005). Understanding and modulating ageing. IUBMB Life.

[6] Stebbing AR (1982). Hormesis--the stimulation of growth by low levels of inhibitors. Sci Total Environ.

[7] Minois N (2000). Longevity and aging: beneficial effects of exposure to mild stress. Biogerontology.

[8] Calabrese EJ, Baldwin LA (2001). Hormesis: u-shaped dose responses and their centrality in toxicology. Trends Pharmacol Sci.

[9] Rattan SI (2001). Hormesis in biogerontology. Crit Rev Toxicol.

[10] Le Bourg E, Minois N, Bullens P, Baret P (2000). A mild stress due to hypergravity exposure at young age increases longevity in Drosophila melanogaster males. Biogerontology.

[11] Le Bourg E, Minois N (1997). Increased longevity and resistance to heat shock in Drosophila melanogaster flies exposed to hypergravity. C R Acad Sci III.

[12] Le Bourg E, Valenti P, Lucchetta P, Payre F (2001). Effects of mild heat shocks at young age on aging and longevity in Drosophila melanogaster. Biogerontology.

[13] Lamb MJ (1964). The Effects of Radiation on the Longevity of Female Drosophila-Subobscura. J Insect Physiol.

[14] Hercus MJ, Loeschcke V, Rattan SI (2003). Lifespan extension of Drosophila melanogaster through hormesis by repeated mild heat stress. Biogerontology.

[15] Cypser JR, Johnson TE (2002). Multiple stressors in Caenorhabditis elegans induce stress hormesis and extended longevity. J Gerontol A Biol Sci Med Sci.

[16] Oh S, Kwon D, Lee E (2011). Cytoprotective activity of elevated static pressure against oxidative stress in normal human fibroblasts. Mol Cell Toxicol.

[17] Alenghat FJ, Ingber DE (2002). Mechanotransduction: all signals point to cytoskeleton, matrix, and integrins. Sci STKE.

[18] Ingber DE (2003). Mechanobiology and diseases of mechanotransduction. Ann Med.

[19] Marshall WF, Kintner C (2008). Cilia orientation and the fluid mechanics of development. Curr Opin Cell Biol.

[20] Barker DJ (1995). The fetal and infant origins of disease. Eur J Clin Invest.

[21] Seckl JR (2001). Glucocorticoid programming of the fetus; adult phenotypes and molecular mechanisms. Mol Cell Endocrinol.

[22] Gluckman PD, Hanson MA, Mitchell MD (2010). Developmental origins of health and disease: reducing the burden of chronic disease in the next generation. Genome Med.

[23] Bateson P, Barker D, Clutton-Brock T, Deb D, D'Udine B, Foley RA, Gluckman P, Godfrey K, Kirkwood T, Lahr MM, McNamara J, Metcalfe NB, Monaghan P (2004). Developmental plasticity and human health. Nature.

[24] Langley-Evans SC (2015). Nutrition in early life and the programming of adult disease: a review. J Hum Nutr Diet.

[25] Vickers MH (2014). Early life nutrition, epigenetics and programming of later life disease. Nutrients.

[26] Soubry A (2015). Epigenetic inheritance and evolution: A paternal perspective on dietary influences. Prog Biophys Mol Biol.

[27] Tarry-Adkins JL, Ozanne SE (2011). Mechanisms of early life programming: current knowledge and future directions. Am J Clin Nutr.

[28] Ganetzky B, Flanagan JR (1978). On the relationship between senescence and age-related changes in two wild-type strains of Drosophila melanogaster. Exp Gerontol.

[29] Minois N, Khazaeli AA, Curtsinger JW (2001). Locomotor activity as a function of age and life span in Drosophila melanogaster overexpressing hsp70. Exp Gerontol.

[30] Chapman T, Partridge L (1996). Female fitness in Drosophila melanogaster: an interaction between the effect of nutrition and of encounter rate with males. Proc Biol Sci.

[31] Tu M-P, Tatar M (2003). Juvenile diet restriction and the aging and reproduction of adult Drosophila melanogaster. Aging Cell.

[32] Dai DF, Chiao YA, Marcinek DJ, Szeto HH, Rabinovitch PS (2014). Mitochondrial oxidative stress in aging and healthspan. Longev Healthspan.

[33] Lopez IA, Acuna D, Beltran-Parrazal L, Espinosa-Jeffrey A, Edmond J (2008). Oxidative stress and the deleterious consequences to the rat cochlea after prenatal chronic mild exposure to carbon monoxide in air. Neuroscience.

[34] Schneider ML, Moore CF, Kraemer GW, Roberts AD, DeJesus OT (2002). The impact of prenatal stress, fetal alcohol exposure, or both on development: perspectives from a primate model. Psychoneuro-endocrinology.

[35] Lithgow GJ, White TM, Melov S, Johnson TE (1995). Thermotolerance and extended life-span conferred by single-gene mutations and induced by thermal stress. Proc Natl Acad Sci USA.

[36] Johnson TE, Cypser J, de Castro E, de Castro S, Henderson S, Murakami S, Rikke B, Tedesco P, Link C (2000). Gerontogenes mediate health and longevity in nematodes through increasing resistance to environmental toxins and stressors. Exp Gerontol.

[37] Mair W, Sgrò CM, Johnson AP, Chapman T, Partridge L (2004). Lifespan extension by dietary restriction in female Drosophila melanogaster is not caused by a reduction in vitellogenesis or ovarian activity. Exp Gerontol.

[38] Barnes AI, Boone JM, Jacobson J, Partridge L, Chapman T (2006). No extension of lifespan by ablation of germ line in Drosophila. Proc Biol Sci.

[39] Gribble KE, Welch DB (2013). Life-span extension by caloric restriction is determined by type and level of food reduction and by reproductive mode in Brachionus manjavacas (Rotifera). J Gerontol A Biol Sci Med Sci.

[40] Harman D (1956). Aging: a theory based on free radical and radiation chemistry. J Gerontol.

[41] Wheeler JC, Bieschke ET, Tower J (1995). Muscle-specific expression of Drosophila hsp70 in response to aging and oxidative stress. Proc Natl Acad Sci USA.

[42] Sorensen JG, Loeschcke V (2002). Decreased heat-shock resistance and down-regulation of Hsp70 expression with increasing age in adult Drosophila melanogaster. Funct Ecol.

[43] Landis GN, Abdueva D, Skvortsov D, Yang J, Rabin BE, Carrick J, Tavaré S, Tower J (2004). Similar gene expression patterns characterize aging and oxidative stress in Drosophila melanogaster. Proc Natl Acad Sci USA.

[44] Landis G, Shen J, Tower J (2012). Gene expression changes in response to aging compared to heat stress, oxidative stress and ionizing radiation in Drosophila melanogaster. Aging (Albany NY).

[45] Kirby K, Hu J, Hilliker AJ, Phillips JP (2002). RNA interference-mediated silencing of Sod2 in Drosophila leads to early adult-onset mortality and elevated endogenous oxidative stress. Proc Natl Acad Sci USA.

[46] Godenschwege T, Forde R, Davis CP, Paul A, Beckwith K, Duttaroy A (2009). Mitochondrial superoxide radicals differentially affect muscle activity and neural function. Genetics.

[47] Martin I, Jones MA, Rhodenizer D, Zheng J, Warrick JM, Seroude L, Grotewiel M (2009). Sod2 knockdown in the musculature has whole-organism consequences in Drosophila. Free Radic Biol Med.

[48] Schriner SE, Linford NJ, Martin GM, Treuting P, Ogburn CE, Emond M, Coskun PE, Ladiges W, Wolf N, Van Remmen H, Wallace DC, Rabinovitch PS (2005). Extension of murine life span by overexpression of catalase targeted to mitochondria. Science.

[49] Fan X, Liang Q, Lian T, Wu Q, Gaur U, Li D, Yang D, Mao X, Jin Z, Li Y, Yang M (2015). Rapamycin preserves gut homeostasis during Drosophila aging. Oncotarget.

[50] Taub J, Lau JF, Ma C, Hahn JH, Hoque R, Rothblatt J, Chalfie M (1999). A cytosolic catalase is needed to extend adult lifespan in C. elegans daf-C and clk-1 mutants. Nature.

[51] Ngo D-N, Kim M-M, Kim S-K (2008). Chitin oligosaccharides inhibit oxidative stress in live cells. Carbohydr Polym.

[52] Pletcher SD, Libert S, Skorupa D (2005). Flies and their golden apples: the effect of dietary restriction on Drosophila aging and age-dependent gene expression. Ageing Res Rev.

[53] Lai C-Q, Parnell LD, Lyman RF, Ordovas JM, Mackay TF (2007). Candidate genes affecting Drosophila life span identified by integrating microarray gene expression analysis and QTL mapping. Mech Ageing Dev.

[54] Merzendorfer H, Zimoch L (2003). Chitin metabolism in insects: structure, function and regulation of chitin synthases and chitinases. J Exp Biol.

[55] Oh S, Lee E, Lee J, Lim Y, Kim J, Woo S (2008). Comparison of the effects of 40% oxygen and two atmospheric absolute air pressure conditions on stress-induced premature senescence of normal human diploid fibroblasts. Cell Stress Chaperones.

[56] Yu S, Jang Y, Paik D, Lee E, Park JJ (2015). Nmdmc overexpression extends Drosophila lifespan and reduces levels of mitochondrial reactive oxygen species. Biochem Biophys Res Commun.

[57] Paik D, Jang YG, Lee YE, Lee YN, Yamamoto R, Gee HY, Yoo S, Bae E, Min KJ, Tatar M, Park JJ (2012). Misexpression screen delineates novel genes controlling Drosophila lifespan. Mech Ageing Dev.

[58] Lee KS, Iijima-Ando K, Iijima K, Lee WJ, Lee JH, Yu K, Lee DS (2009). JNK/FOXO-mediated neuronal expression of fly homologue of peroxiredoxin II reduces oxidative stress and extends life span. J Biol Chem.

[59] Ha EM, Oh CT, Bae YS, Lee WJ (2005). A direct role for dual oxidase in Drosophila gut immunity. Science.

[60] Dennis G, Sherman BT, Hosack DA, Yang J, Gao W, Lane HC, Lempicki RA (2003). DAVID database for annotation, visualization, and integrated discovery. Genome Biol.

[61] Huang W, Sherman BT, Lempicki RA (2009). Systematic and integrative analysis of large gene lists using DAVID bioinformatics resources. Nat Protoc.

